# Quantitative downhill skiing technique analysis according to ski instruction curricula: A proof-of-concept study applying principal component analysis on wearable sensor data

**DOI:** 10.3389/fbioe.2022.1003619

**Published:** 2022-09-27

**Authors:** Daniel Debertin, Felix Wachholz, Ralf Mikut, Peter Federolf

**Affiliations:** ^1^ Department of Sport Science, University of Innsbruck, Innsbruck, Austria; ^2^ Institute for Automation and Applied Informatics, Karlsruhe Institute of Technology, Karlsruhe, Germany

**Keywords:** alpine skiing, biomechanics, inertial measurement unit, principal component analysis, coordination, human movement, kinematics, winter sport

## Abstract

*Downhill skiing technique* represents the complex coordinative movement patterns needed to control skiing motion. While scientific understanding of skiing technique is still incomplete, not least due to challenges in objectively measuring it, practitioners such as ski instructors have developed sophisticated and comprehensive descriptions of skiing technique. The current paper describes a 3-step proof-of-concept study introducing a technology platform for quantifying skiing technique that utilizes the practitioners’ expert knowledge. The approach utilizes an inertial measurement unit system (Xsens™) and presents a motion analysis algorithm based on the Principal Movement (PM) concept. In step 1, certified ski instructors skied specified technique elements according to technique variations described in ski instruction curricula. The obtained data was used to establish a PM-coordinate system for skiing movements. In step 2, the techniques *parallel* and *carving turns* were compared. Step 3 presents a case study where the technique analysis methodology is applied to advise an individual skier on potential technique improvements. All objectives of the study were met, proving the suitability of the proposed technology for scientific and applied technique evaluations of downhill skiing. The underlying conceptual approach - utilizing expert knowledge and skills to generate tailored variability in motion data (step 1) that then dominate the orientation of the PMs, which, in turn, can serve as measures for technique elements of interest - could be applied in many other sports or for other applications in human movement analyses.

## Introduction

Downhill skiing is a very popular but also very demanding sport (Hébert-Losier et al., 2014)—particularly in terms of coordinative and adaptive motor control skill requirements. *Skiing technique* represents the complex coordinative movement pattern needed to not only control and direct the large forces acting on and in the skier’s body (LeMaster, 2010), but also needed to cope with changing environmental conditions such as varying snow type, visibility, slope gradient, terrain unevenness etc. (Skilehrerverband, 2019).

Previous research, where skiing technique assessments played a role, was often motivated by the goal of understanding injury mechanisms (Urabe et al., 2002; Krosshaug et at., 2007; Pellegrini et al., 2018; Promsri et al., 2019), prevention of injuries (Spörri et al., 2017), improvement of racing performance (Roetenberg et al., 2009; Reid, 2010; Federolf, 2012; Hébert-Losier et al., 2014; Robert-Lachaine et al., 2017), and some studies assessed fundamental (bio-)mechanical aspects of skiing (Müller, 1994; Müller and Schwameder, 2003; Müller et al., 2005;, 2010; Klous et al., 2012; Meyer, 2012; Lind and Sanders, 2013). Despite these numerous investigations, the scientific understanding of many aspects of skiing technique is still incomplete. Moreover, the complexity of the skiing movements and the inhospitable environment pose particular challenges for adequate measurement technologies and hamper quantitative evaluation (Klous et al., 2010).

In contrast, practitioners, e.g. the ski schools or ski instructor associations, have developed structured and comprehensive descriptions of skiing technique. In particular, many ski instructor associations contrived instruction curricula to teach beginners how to ski (Skischulverband, 2015; Skilehrerverband, 2019), in most cases with clearly defined milestones, e.g. the “parallel turn” (side-skidding with parallel ski control) and the “carving turn” (skiing on the ski edges without side-skidding). Moreover, the curricula also describe specific technique modifications/elements, for example, skiing in a forward or in a backward leaning position, skiing with or without pronounced vertical motion, turning with inward leaning versus turning with an upright upper body, etc. (LeMaster, 2010). Licensed ski instructors are not only required to recognize the techniques and technique elements in their clients’ skiing to advise on potential improvements, they are also required to be able to demonstrate them themselves. Unfortunately, the expert knowledge that the practitioners have developed so far remains a qualitative description of skiing technique and researchers were only marginally able to utilize the expert knowledge of practitioners (Loland, 2009). The vision for the current project was therefore to establish a measurement and data analysis platform that allows to quantitatively assess skiing technique in such a way that it utilizes and is compatible with the approach and knowledge of expert ski practitioners.

Wearable sensor technology based on inertial measurement units (IMUs) (Kröll, et al., 2015; Fasel, et al., 2016) provides a first building block for the envisioned technology platform. Specifically, we utilized the commercially available Xsens™ system which had already been tested and validated for human movements recording for laboratory (Al-Amri, et al., 2018; Teufl et al., 2019) as well as for on-snow environments (Krüger and Edelmann-Nusser, 2010; Supej, 2010). IMU technology offers the advantage of instant and direct data availability for processing (Spörri, 2012), in contrast to other data acquisition technologies, for instance, the optical video reconstruction from panning, tilting and zooming cameras (Mössner et al., 1996; Nachbauer et al., 1996) or from fixed camera systems such as Vicon™ (Klous et al., 2010; Spörri et al., 2016) or Qualisys™ (Reid, 2010). The second building block for the envisioned technology platform is a data analysis algorithm based on a principal component analysis (PCA) (Troje, 2002; Daffertshofer et al., 2004). The specific approach introduced in the current paper is conceptually based on earlier studies (Federolf et al., 2014; Gløersen et al., 2018), but does add new conceptual ideas.

The challenge addressed in the current study is the establishment of a procedure to utilize expert knowledge of the practitioners—in our case skiing instructors but our approach could similarly be utilized in other sports with practical expert knowledge on technique—to provide quantifiable data for the practitioners’ qualitative descriptions of technique. In contrast to previous studies, the current study tailored the PCA output to specific technique elements of interest by beforehand creating an additional dataset whose variance is purposefully manipulated through having skiing instructors demonstrate specific technique features. Through this procedure, we can for the first time quantitatively assess skiing technique in a manner consistent with the technique descriptions of skiing experts. In summary, the current study represents a three-step proof-of-concept study. The goal of the first step was to obtain—through a PCA based on wearable sensor data—a coordinate system for skiing movements, which aligns with the movement descriptions used in the Austrian ski instruction curriculum (Skischulverband, 2015). Thus, we obtain objective measurement scales for skiing technique elements. The goal of the second step was to apply this movement evaluation system in an assessment of differences between the skiing techniques “parallel turn” and “carving” (Skischulverband, 2015). The goal of the third step was to demonstrate practical applicability of our method through comparing the technique of a ski instructor aspirant (good skier, but has not passed the instructor license exams yet) with the techniques of certified ski instructors.

## Materials and methods

### Participants

Eight experienced and highly educated ski instructors (3 female, five male; *M* = 27.0 years, *SD* = 3.0) participated in the study. The main inclusion criterion was an active ski instructor license: half of the participants held a national and the other half a regional instructor license. Further inclusion criteria were age above 18, skiing experience of more than 10 years and more than 30 seasonal skiing days. Exclusion criteria were any recent injuries which might influence skiing abilities. The aspirant recruited for the third step of the study fit the same inclusion criteria with the exception of the active instructor license. All participants were informed about the background and the purposes of the study and provided written consent. The study was approved by the Board for Ethical Questions in Science of the University of Innsbruck (certificate 55/2019).

### Study design

**FIGURE 1:**
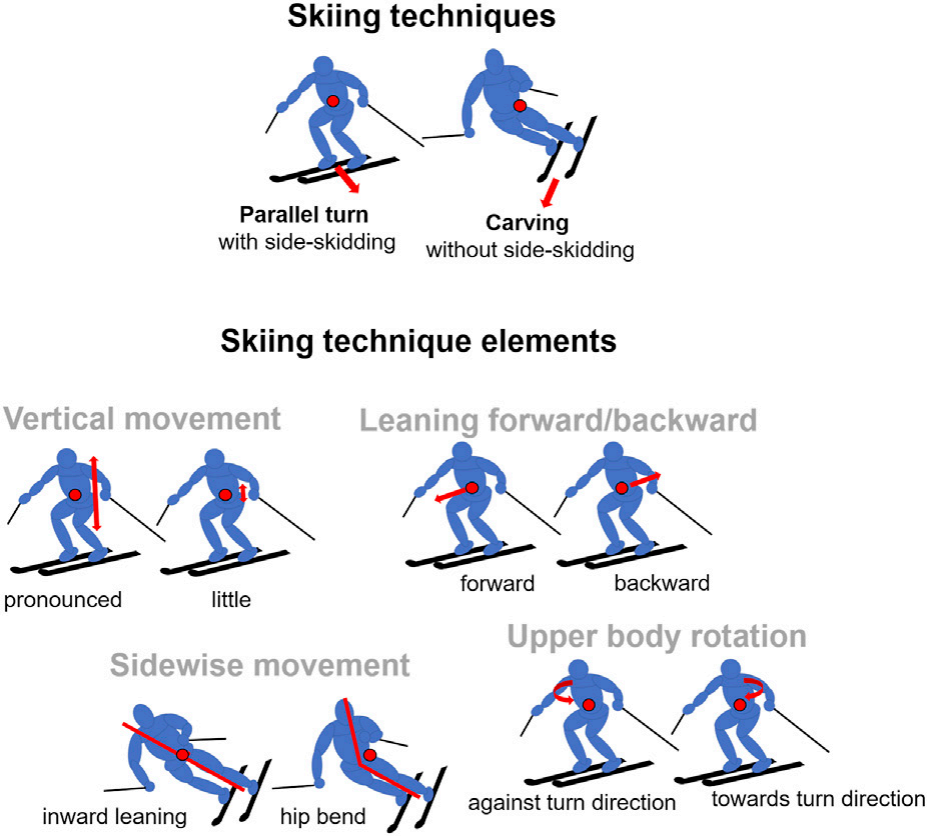
Schematics illustrating the main techniques of parallel and carving turns, as well as the involved skiing technique elements based on the descriptions in the Austrian ski instruction curriculum (Skischulverband, 2015) and demonstrated by the ski instructors within the study.

A coordinate system aligning with technique elements as described in the skiing curricula (step 1) can be obtained through a PCA when tasking the ski instructors with modifying their skiing according to eight distinct technique elements. Specifically, we instructed the skiers to use parallel turns as the base technique and to then modify this technique by forward versus backward leaning, pronounced versus little vertical movement, inward leaning versus hip bending, and rotating the upper body towards versus against the turn direction (Figure 1). The testing order of these four pairs of opposing technique instructions was randomized between participants. In addition, for step 2, the instructors were asked to ski the techniques parallel turn and carving turn (Figure 1) precisely according to the descriptions in the Austrian ski instruction curriculum (Skischulverband, 2015). Further instructions were to ski with equal turn radii and to aim for a smooth and natural movement execution. Each technique and each technique element were skied in one separate run of at least seven complete turns. Prior to testing, skiers had performed several warm-up runs. Before each run, sensors were calibrated by walking a short distance in ski boots over a flattened area of the ski piste and standing in neutral position. The measurements were carried out at the ski resorts Axamer Lizum and St. Christoph am Arlberg, Austria on even and moderately steep slopes (*M* = 23.1% gradient, *SD* = 0.6). The testing period was half a day for each participant. Weather and snow conditions were similar and allowed for easy controllable skiing.

### Data acquisition

**FIGURE 2:**
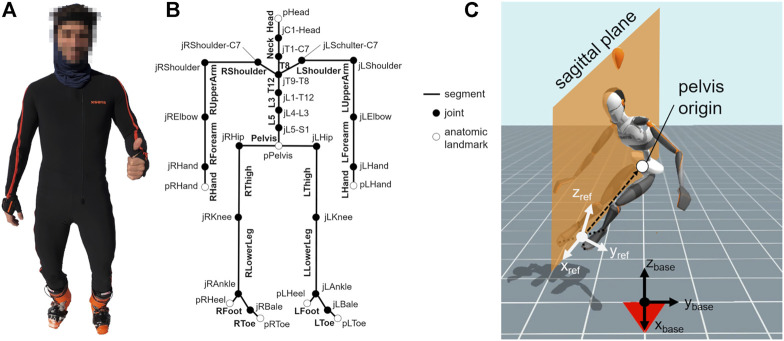
**(A)** Volunteer wearing the Xsens™ suit for skiing: sensors on the feet were attached to the ski boots from the outside; **(B)** body model with extracted reference points for body segment positions; **(C)** reconstructed avatar (adopted from Xsens™ software) with reference coordinate system.

Kinematic data was recorded using Xsens^TM^ MVN Technology (Xsens Technologies B.V., Enschede, Netherlands). The hardware (Firmware Version 1.2.0) consisted of 17 inertial measurement units (gyroscopes, accelerometers and magnetometers) operating at 240 Hz, which were placed at prescribed body positions within a tight Lycra suit (Figure 2A). Foot sensors were placed on the outside of the ski boots above the foot arch, wrapped in foil to protect them against humidity and cold, and attached with duct tape. The Xsens^TM^ software (Version 2019.2) postprocesses the recorded sensor raw data by combining all available information using Kalman filters and biomechanical constraints. The calibration process ensures the sensors’ position alignment with the implemented human model (Figure 2B), which is based on 23 rigid segments. The software outputs 3D segment and estimated center of mass (COM) coordinates in relation to the pelvis origin. In order to visually compare reconstructed poses with the original movement, every trial was additionally filmed using a GoPro Hero 8 camera (GoPro Inc., San Mateo, United States).

### Data analysis

**FIGURE 3:**
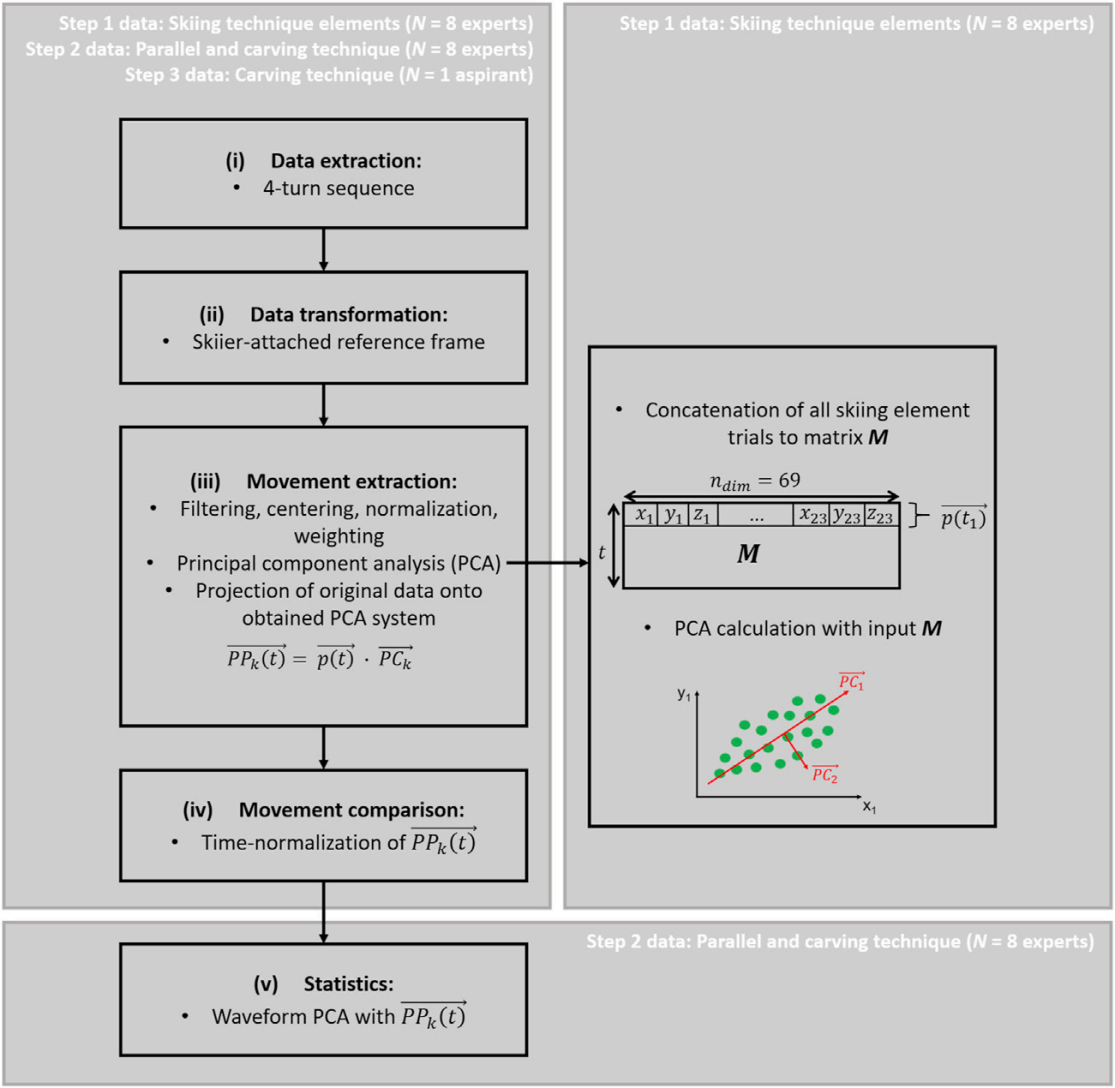
Flowchart of data analysis steps from data extraction of four consecutive turns, to transformation into skier-attached reference frame, movement extraction by principal component analysis (PCA), comparison through time-normalization and statistical evaluation. The PCA is performed for the skiing technique element trials (step 1 data) and the skiing technique trials (step 2 and 3 data) are projected onto the obtained PCA system.

The current study analyzed 3D segment position data (represented by segment origin: proximal joint position). Data processing was coded in MATLAB R2019b (The MathWorks, Natick, Massachusetts, United States). Data analysis (Figure 3) consisted of the five main steps: (i) identification of turn cycles and extraction of four consecutive turns; (ii) transformation of position data into a skier-attached reference frame; (iii) partial movement extraction by PCA; (iv) time-normalization of the turn data through interpolation; (v) statistical analysis to assess differences between carving and parallel turn waveforms. The following paragraphs provide details about these analysis steps.

(i) The turn sequences were determined through first setting up an interim reference frame with its origin in the midpoint of all toe and heel markers; its x-axis pointing towards the midpoint between the toe markers; the z-axis was the Xsens™-z-axis, which points vertically upwards against gravity; and the y-direction resulted from a cross product of x and z. Within this system, the transition between ski turns was determined as the time point when the COM’s y-coordinate was zero (i.e. when the skier was upright on the skis). From each trial, four consecutive turns, a left-right-left-right turn sequence, were extracted for analysis.
(ii) The skier-attached reference frame (Federolf et al., 2014) was then obtained through a coordinate rotation around x, such that the x-y-plane contained the center of the pelvis (Figure 2C). Thereby the resultant coordinate system inclines with the skier into the turn.
(iii) The time series of the 3D segment positions of the 4-turn sequence of each trial were then filtered with a 4th-order, 50 Hz low-pass Butterworth filter, centered by subtracting the mean posture of the skier, normalized to mean Euclidian distance (Federolf et al., 2008; Federolf et al., 2013) to allow comparisons between subjects, and weighted using De Leva’s relative segment masses (De Leva, 1996). Then, the trials in which the skiers had performed the eight distinct technique elements (step 1), were concatenated to form a single input matrix for the PCA [(8 participants * 8 trials * time points) x (23*3 segment positions)]. The data from the parallel and carving turns (step 2) and from the case study (step 3) were not used for calculating the PCA, but were later projected onto the PCA system obtained from step 1. The data pre-processing steps and the PCA calculation, as described in the current paragraph, were performed using the PManalyzer, a publicly available software toolbox (Haid et al., 2019). The PCA provides a new coordinate system spanned by the eigenvectors (PC-vectors) of the covariance matrix. Each PC-vector represents a specific pattern, how a given body configuration deviates from the mean posture. We refer to these partial movements represented by each PC-vector as “principal movements” (PMs) (Federolf et al., 2013; Federolf, 2016). The first few PMs explain the greatest amount of variability in the data set, and since we produced large variability by instructing the skiers to ski specific technique elements in opposite extremes (step 1), we achieve an alignment of the PC-vectors with the given technique specifications. We can visualize each PM as animated stick figures by a retransformation onto the original system (Supplementary Material). By transforming the original data onto the PMs, time series of principal positions (PP(t)s) are obtained. The PP(t)s provide measures for the skiers’ movements expressed according to the PMs. Technique differences between parallel and carving turns could thus be quantified through projecting these turns also onto the PM-coordinate system.
(iv) As a last data processing step, the PP(t) obtained from the 4-turn sequences were time-normalized by interpolation to 100 data points per left-right turn sequence. Thereby, comparisons between different skiing technique elements, different techniques (parallel vs. carving) and different skier expertise (instructor vs. aspirant) were enabled.
(v) The time-normalized PP(t) waveforms could then be averaged for graphical display and statistically tested for differences between the parallel and the carving technique.

### Statistics (parallel versus carving skiing technique)

To determine technique differences between parallel and carving turns, we assessed differences in the shape of the PP(t) waveforms. Thereto, the PP(t) graphs were submitted to a waveform-PCA, i.e. inputs were the 100-point waveform shapes (Mohr et al., 2021). The scores of the first component, i.e. the main feature producing waveform variability, served as dependent variable and was statistically evaluated.

All statistical calculations were conducted using the software Jamovi 1.1.9.0 (The jamovi project, 2021). The Shapiro-Wilk test confirmed normality for all PP(t) scores. Therefore, we report the results of paired t-tests with Cohen’s *d* quantifying the effect size. Due to the small sample size (*N* = 8) we further corroborated all statistically implied conclusions through the corresponding non-parametric tests (Wilcoxon signed-rank test), for which we found no discrepancies to the t-test results. Additionally, a Holm-Bonferroni-correction (Holm, 1979) was applied to account for the fact that six t-tests (we considered the first six PP(t)s since they were visibly affected by the technique elements and represented 99% of the postural variance) were conducted. In all tests we used *α* = 0.05 as the base threshold for statistical significance. We refer to effect sizes of *d* > 0.8 as strong effects (Cohen, 1992).

### Case study of ski instructor aspirant

The volunteer was asked to perform carving turns according to the skiing curriculum (Skischulverband, 2015) on the same slope where the ski instructors had conducted their trials. Similar to step 2, the data was projected onto the eigenvectors obtained from the analysis of step 1. PP(t) results were graphically visualized and compared to the mean trajectories of the certified ski instructors.

## Results

### The PM-coordinate system for skiing technique

**FIGURE 4:**
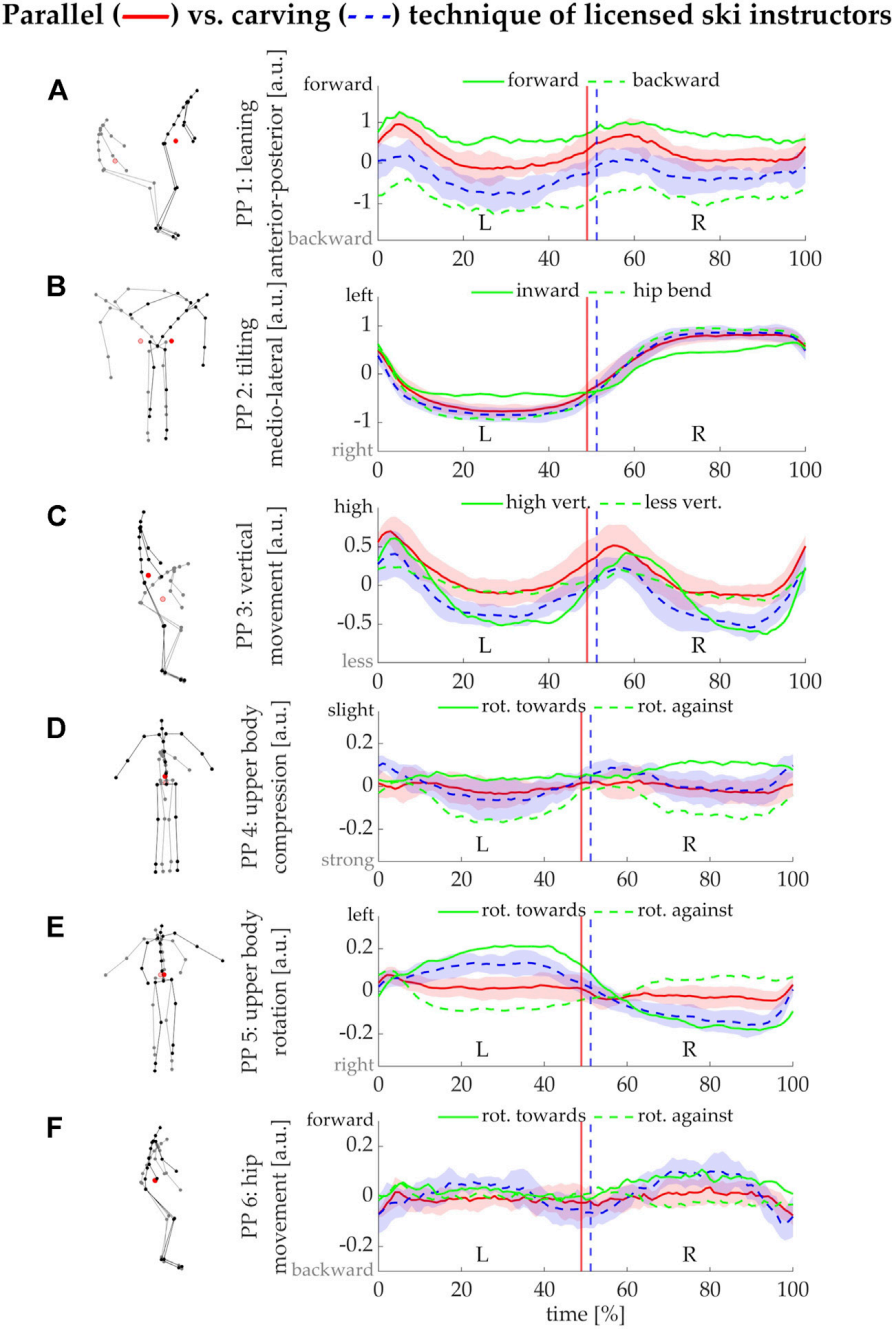
Left: stick figure representation of the first six principal movements (PMs). Right: time series representation for each principal movement position PPk(t) [k = 1.6] **(A–F)** interpolated to 100% of a left(L)-right(R) ski turn cycle. For each PM, the instruction trials that caused the largest differences in the PP(t)—e.g., for PM1 the instructions to lean forward or backward—are displayed as continuous and broken green lines (means over all turn cycles of all volunteers). The red and blue lines and shaded areas represent the mean and standard deviations obtained from the parallel and carving turns of all the volunteers, respectively. Vertical lines indicate the mean time point of transition from L to R turn.

**FIGURE 5:**
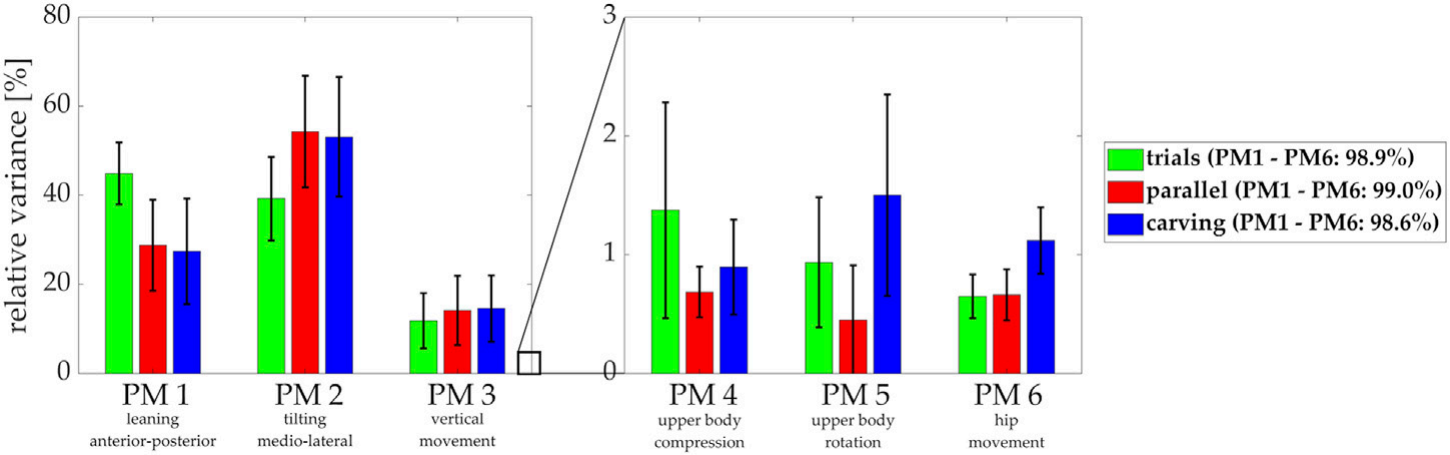
Relative variances explained by the first six principal movements (PMs) for the trials with instructed technique variations (green bars) [this is the data for which the PCA was calculated in step 1] and relative variances for the parallel (red) and carving turns (blue) analyzed in step 2 [these data were projected onto the PC-eigenvectors obtained in the analysis of step 1].

The coordinate system produced by the PCA, particularly axes PM1, PM2, PM3, and PM5, aligned well with the changes in posture produced by the specific technique instructions. The first eigenvector (PM1) captured changes in posture associated with anterior-posterior body positioning (stick figure in Figure 4A). Accordingly, PP1(t) can serve as a measure for quantifying forward (continuous green line in Figure 4A) or backward leaning (broken green line) in the skiing technique. PM1 quantified 44.9% of all postural variances observed in the specific technique trials (green bars in Figure 5). PM2 captured a medio-lateral tilting (moving away from the sagittal plane) of the upper body and, accordingly, the technique instructions of inward leaning as opposed to hip bending (green lines in Figure 4B) produced the largest differences in PP2(t) waveform shape. PM2 represented 39.2% of the postural variances of the technique trials. PM3 represented 11.8% of the variance and captured knee flexion together with a crouching motion of the upper body. The instruction to ski with large versus little vertical movement produced the largest differences in the PP3(t) graphs (Figure 4C). PM4 (1.4% of postural variance) captured a change in posture that appeared as upper body compression and arm motion in the stick figure representation. PM4 can be interpreted as a residual posture change arising from the linearization of anatomical movements. The instruction pair of rotating with as opposed to against the turn produced the largest differences in the PP4(t) graphs (Figure 4D). PM5 (0.9% of postural variance) captured upper body rotations and, accordingly, the instruction to rotate with or against the turn produced the largest differences also in PP5(t) (Figure 4E). Finally, PM6 captured a hip positioning and slight crouching, but represented only 0.6% of the variance. PP6(t) also showed the largest differences for the instructions of rotating with versus against the turn (Figure 4F).

### Parallel and carving techniques assessed in the PM-coordinate system

The first six PMs together covered 99.0 and 98.6% of the postural variance of the parallel and carving techniques, respectively (Figure 5). Interestingly, for both techniques the PM2 movement (medio-lateral tilting) now contributed more to the overall postural variance than PM1 (anterior-posterior leaning).

Differences in the PP(t)-waveform shape between the techniques appeared for PM1, PM3, PM4, PM5, and PM6, demonstrating that carving involves more backward leaning (PM1: *t* (7) = 4.3, *p* = 0.003, *d* = 1.53) and overall a more crouched position (PM3: *t* (7) = 4.8, *p* = 0.002, *d* = 1.68) than the parallel turn technique. Also, carving is performed with rotating the upper body with the turn, while the parallel turn shows upper body rotation against the turn (PM5: *t* (7) = 6.0, *p* < 0.001, *d* = 2.13). Lateral tilting (PM2) did not differ significantly between techniques (*p* = 0.363). The carving technique also showed more movement in PM4 (*t* (7) = 3.0, *p* = 0.019, *d* = 1.07) and PM6 (*t* (7) = 3.1, *p* = 0.018, *d* = 1.08) compared to the parallel turn, for which a neutral positioning with relatively little changes throughout the turns were found in both movement components.

### Case study: Individual skiing technique assessment

**FIGURE 6:**
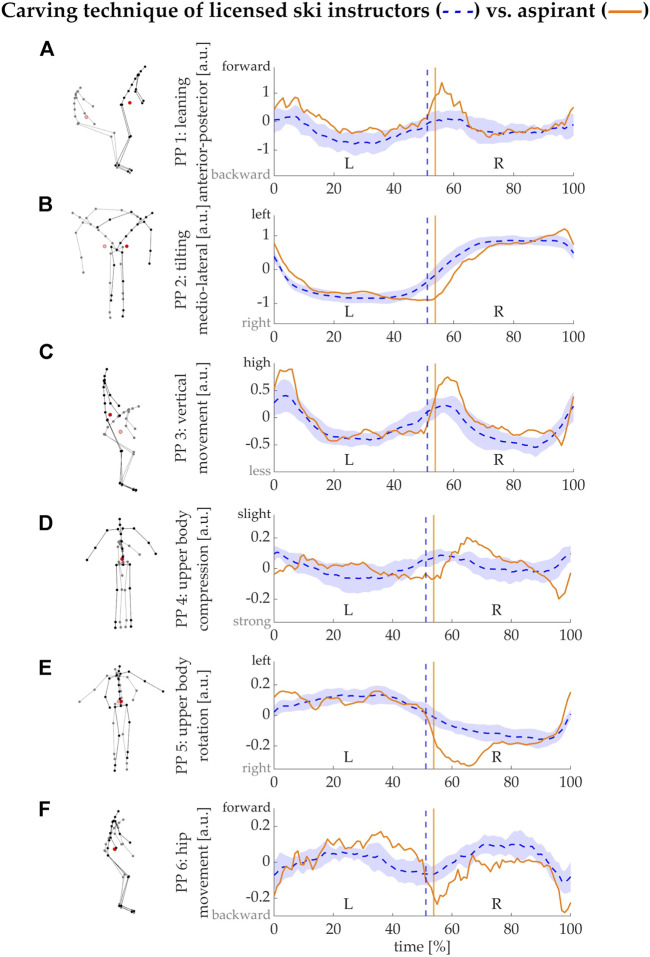
Case study: comparison of the individual technique of a specific skier with reference data from the ski instructors. PM1-6 **(A–F)**, visualized by the stick figures and displayed by the PP(t) series are adopted from Figure 4 for all carving turns of the skiers with active ski instructor license (blue lines and shaded areas for mean and standard deviation). The recorded carving turns of the aspirant are projected onto the same PM-coordinate system (orange lines).

Figure 6 visualizes the assessment of the individual technique of the volunteering instructor aspirant in comparison with the combined carving turn data of the licenced instructors. We can provide the feedback, that the candidate showed more vertical motion (Figure 6C) in combination with more forward movement (Figure 6A) when initializing the new turns compared to the reference skiers. Particularly in the first half of the right turn, pronounced rotation of the upper body is visible (Figure 6E), which the peers do not show. Also, more pronounced hip movements are visible (Figure 6F). Overall, due to the pronounced body actions (vertical motion, rotating into the turn) the movement appears jerkier compared to the relatively smooth motion seen in the instructor data. Based on these particular turns, we would recommend to the aspirant to practice carving turns with less pronounced vertical motion during turn initiation (this will likely also reduce the pronounced forward motion in PM1) and to practice with less upper body rotation.

## Discussion

The objectives of the current proof-of-concept study were 1) to develop a measurement methodology for skiing technique based on the approaches and technique descriptions found in ski instruction curricula; 2) use this methodology to evaluate differences between the parallel turn and carving turn techniques; 3) in a case study, evaluate technique differences between an individual skier and reference data obtained from ski instructors. We accomplished these goals through performing a PCA on data obtained in trials where we asked experts to perform specific technique elements. The results shown in the current paper demonstrate and prove suitability of the conceptual approach for the purposes of technique evaluations in downhill skiing.

### Conceptual considerations

This approach is not limited to skiing, but could be applied in many other sports where qualitative technique descriptions are available. It could also be applied in other contexts of human movement analysis to quantify specific, so far only qualitatively described behavior. Examples could be quantification of body language in psychology, quantification of movement patterns in work place environments, or automated behavior recognition problems in human-robot interactions.

### Technique elements in skiing

Variations in the forward-backward positioning of the body over the ski is a technique variation that skilled ski instructors can demonstrate routinely and it leads to substantial variance in the overall body posture. Therefore, not surprisingly, this technique element defined the first principal component eigenvector and thus PM1. Within the ski turns, we observed that during the early phase of the turn (turn initiation), a forward movement can be observed in all trials. During the second half of the turns (steering phase) the skiers’ bodies shifted slightly backwards. These findings are consistent with ski instruction curricula (Skischulverband, 2015; Skilehrerverband, 2019). The ability to quantify forward-backward leaning provides several opportunities for future research, for example, extensive backward leaning is frequently observed in novice skiers and is often considered a mistake since backward positioning makes control of the skiing motion more difficult (Skilehrerverband, 2019). Our methodology for studying skiing technique might make it possible to better understand the mechanisms leading to backward leaning in novices and might reveal which instructions or exercises could help novices to better gain control over their positioning. Additionally, backward leaning is also relevant from an injury mechanism and prevention perspective, since it increases the moments of force acting on the knee and increases strains on the anterior cruciate ligament (ACL) (Eberle et al., 2019; Raschner et al., 2001; Yoneyama and Okamoto, 2001; Yu et al., 2016; Zago et al., 2017a; Yoshioka et al., 2017; Zago et al., 2017;, 2019; Färber et al., 2019; Heinrich; Werner et al., 2021; Federolf, 2019). In several situations, backward leaning is an important contributing factor to an elevated injury risk (Bere, et al., 2011; Brodie et al., 2008; Heinrich et al., 2022).

The instruction pair “inward leaning into the turn” versus “hip bending” produced the largest differences visible in PM2. Contrary to the situation in PM1, however, the postural variance was here not mainly a consequence of the given instruction. Instead, large postural variance is produced by the skiing movements themselves during the left-right turn sequence, which require a leaning to the left and right, respectively. When explicitly instructed to lean into the turn and not to hip-bend, then the ski instructors were able to demonstrate this technique variation clearly enough to be detectable in PM2, but they still had to lean to the left and right, as is visible in Figure 4B.

The instructions to show pronounced or little vertical movement are another set of technique variations that ski instructors can routinely demonstrate. Accordingly, differences between these trials are clearly visible in PM3, which mostly captured the vertical motion. The corresponding graph in Figure 4C suggests, that the instructors could substantially reduce their vertical motion when asked to do so, however, in the data obtained in the current study, the skiers still showed some upward motion in the turn initiation phase. Mechanically, the vertical motion is believed to regulate the load/forces onto the skis. Therefore, future research where our technique measurements are combined with pressure insoles in the ski boots or with force plates in the ski binding would be interesting.

The instruction pair to rotate the upper body towards versus against the turn influenced all three remaining PMs (Figures 4D–F) analyzed in the current study. This was expected, since PCA produces a linear coordinate system and consequently, any rotation will necessarily affect several (at least two) PMs. PM5 is probably the best suited as a scale for this technique variation, since on the one hand, the stick figure representation comes closest to the expected posture variation, and on the other hand, the opposite instructions led to opposite behavior in the PP5(t) graph (Figure 4E).

In summary, all investigated technique variations demonstrated by the ski instructors volunteering in our study led to measurable differences in the PP(t) trajectories calculated based on this data. Investigation of more technique elements would be possible through analogue procedures.

### Differences between the parallel and carving technique

A methodologic point to discuss before evaluating technique differences between parallel and carving turns is the question, whether it is justified to project data obtained from “carving” onto coordinate axes obtained from technique variations based on the “parallel turn” technique. Our data suggest that it was justified, since even for the carving turns, when projected onto our six PMs, 98.6% of the entire postural variance was explained (Figure 5). In comparison, for the parallel turns 99.0% of postural variance was explained, i.e. only marginally more. For both skiing techniques, the first six PMs together provided very close approximations of the skiers’ movements.

### Case study: Evaluation of an individual’s technique

The case study results demonstrate applicability of the presented technique measurement approach for providing individualized feedback to skiers. The outlined case, an aspirant for the ski instructor exams, is an example where such feedback would be particularly useful: perception of one’s own skiing can be misleading. Aspirants therefore often require and depend on the feedback of experienced instructors when they train required technique forms. Objective feedback on one’s own technique through our approach and thus independent of an expert observer could create more opportunities for practice. In addition to the feedback in terms of the technique variations defined in ski instruction curricula, as described in the current paper, the PM approach can also provide feedback in form of animated stick figures. This might be useful, both, when the definitions of the specific technique elements are not entirely clear to an aspirant, or generally in ski instructor education to better recognize technique features in a skier.

### Limitations

The small number of volunteers (*N* = 8) is a limitation of the current study. Recruitment into the study is limited, on the one hand, by the requirement of finding certified experts to volunteer; on the other hand, it is also a result of environmental conditions since unsuitable weather or snow conditions precluded testing on some days. Another limitation is that the quality of the results in the current study depends on the expert skiers’ ability to demonstrate the instructed technique elements. In our opinion, the data suggests good agreement of the skiing techniques among the experts, suggesting that they were all able to properly execute the instructed techniques. It should be noted here, that all expert skiers in the current study were Austrian ski instructors. Skiing curricula and instructor education differ between countries, experts from other countries might therefore demonstrate the techniques differently or might differ in their execution of the parallel and carving turns.

Technical limitations arise from the chosen hardware and measurement principles. Particularly drift in the data is an issue. To minimize drift, recalibration was done after every downhill run. For the analysis of postural movements as conducted in the current study the Xsens^TM^ device provided sufficient accuracy, however, it was not possible to extract the skier trajectory in an external coordinate system. For that purpose, combinations of an IMU-based sensor system with a global positioning system is likely necessary.

Regarding limitations in the data analysis algorithms, it should be noted, that PCA provides a linear coordinate system. Many forms of body segment movements, particularly rotations, project onto several PC-vectors. Specific PMs can serve as measures or as approximations for specific technique elements—as the current study shows—but they should not be misunderstood as the technique elements themselves.

## Conclusion

The current proof-of-concept study accomplished a so far unsolved technological challenge: “how can skiing technique be quantified in accordance with experts’ qualitative descriptions of skiing techniques?“. Our solution provides objective measures for skiing technique, in which we utilized the expert knowledge of ski experts (ski instructors) and skiing curricula. We analyzed technique differences between two well-defined skiing techniques, parallel turns and carving, and we present a case study, how individual technique could be compared to reference data from other skiers to provide individualized feedback.

## Data Availability

The raw data supporting the conclusions of this article will be made available by the authors, without undue reservation.
